# Geriatric Assessment with Geriatric-8, Body Weight Loss, and Bioelectrical Impedance Analysis in Older Patients with Lung Cancer: A Single-Center Retrospective Study

**DOI:** 10.31662/jmaj.2025-0395

**Published:** 2025-12-19

**Authors:** Tomonori Hirashima, Naoki Yoshimoto, Yoshitaka Fujii, Satoru Yamamoto, Yuhki Takahashi, Masaki Ninomiya, Keita Mizukoshi, Sora Obata, Yusuke Watari, Akira Todoriki, Erika Matsui, Atsuhito Hikiishi, Eriko Tani, Kenji Nakahama, Hidekazu Suzuki, Nobuhiro Izumi, Kenichi Minami, Kazuto Hirata

**Affiliations:** 1 Department of Thoracic Oncology, Ishikiriseiki Hospital, Higashiosaka, Osaka Prefecture, Japan; 2 Respiratory Medicine, Ishikiriseiki Hospital, Higashiosaka, Osaka Prefecture, Japan; 3 Thoracic Surgery, Ishikiriseiki Hospital, Higashiosaka, Osaka Prefecture, Japan; 4 Physical Therapy, Ishikiriseiki Hospital, Higashiosaka, Osaka Prefecture, Japan; 5 Osaka Habikino Medical Center, Department of Thoracic Oncology, Habikino, Osaka Prefecture, Japan; 6 Department of Thoracic Surgery, NHO Osaka Minami Medical Center, Kawachinagano, Osaka Prefecture, Japan

**Keywords:** lung cancer, geriatric assessment, geriatric-8, body weight loss, skeletal muscle mass index, extracellular water-to-total body water ratio, sarcopenia, cancer cachexia

## Abstract

**Introduction:**

There is no standard geriatric assessment (GA) for patients aged ≥65 years with lung cancer (hereafter referred to as patients). This retrospective study evaluated whether GA could be achieved by combining Geriatric-8 (G8) score (G8s), body weight loss (BWL) of more than 5% (5%BWL), and bioelectrical impedance analysis (BIA).

**Methods:**

This study included patients who underwent G8 screening, BIA (measuring skeletal muscle mass index [SMI] and extracellular water-to-total body water ratio [ECW/TBW]), and physical function tests before treatment at our hospital between March 1, 2023, and December 31, 2024. Patient clinical records were reviewed to collect baseline data. Statistical analyses were conducted using R (version 4.1.1).

**Results:**

A total of 120 patients were analyzed. We found the following significant associations: G8s ≤14.0 and 5%BWL were associated with advanced-stage disease; G8s >14.0 and SMI ≥cut-off value (CV) with higher body mass index; ECW/TBW ≥0.4 (0.4 ECW/TBW) with aging and poor performance status; 5%BWL with lower maximum lower leg calf circumference (MLLCC); SMI ≥CV with higher maximum handgrip strength (MHGS) and MLLCC; 0.4 ECW/TBW with lower MHGS, gait speed, and five-time sit-to-stand performance. The multivariate analysis confirmed significant associations: G8s ≤14.0 was associated with cancer cachexia; SMI < CV and 0.4 ECW/TBW were associated with sarcopenia, and 0.4 ECW/TBW was associated with physical function decrease, as indicated by a Short Physical Performance Battery score of ≤9. Patients with G8s ≤14.0, 5%BWL, or 0.4 ECW/TBW had shorter survival durations than their respective counterparts. Patients were classified into three frailty categories (none, mild combined with moderate, severe) based on a combination of four factors (G8, BWL, SMI, and ECW/TBW) and had distinct survival curves.

**Conclusions:**

The combination of these four factors offers a simple and objective approach for GA in patients.

## Introduction

As of 2021, approximately 761 million individuals aged ≥65 years were living worldwide, and this number is projected to increase to 1.6 billion by 2050 ^(1)^. With advancing age, the ability of older adults to recover from physiological and environmental stressors decreases owing to age-related physical changes. This increased vulnerability, also known as frailty, is common among older adults and is associated with increased health care utilization and adverse health outcomes. Frailty is defined as a state of increased susceptibility to poor resolution of homeostasis after a stressor event, thereby increasing the risk of adverse outcomes, which include falls, delirium, and disability ^(2)^. In older adults with cancer, frailty is associated with increased all-cause mortality over 5 years ^(3)^.

According to 2019 statistics from the National Cancer Center Hospital, the incidence rates of lung cancer were 85.1%, 51.5%, and 16.4% among patients aged ≥65, ≥75, and ≥85 years, respectively, with patients aged ≥75 years now accounting for more than half of all lung cancer cases ^(4)^.

The Japan Clinical Oncology Group (JCOG) Committee on Older Patients’ Research Policy ^(5)^ categorizes older patients into three groups: (1) fit patients who can receive the same treatment as healthy non-older patients; (2) vulnerable patients who cannot receive the same treatment as healthy non-older ones but can tolerate certain treatments; and (3) frail patients for whom only best supportive care or palliative care appears appropriate. However, methods for classifying patients into these categories are currently lacking.

Functional assessment is essential for identifying frailty risk in older patients. The Geriatric-8 (G8) is considered a practical and effective geriatric assessment (GA) tool owing to the (60%-90%) frailty prevalence in older patients with cancer ^(6), (7), (8)^.

The cluster randomized trial for elderly NSCLC patient using geriatric assessments (ENSURE-GA) study ^(9)^, presented at the 2024 American Society of Clinical Oncology annual meeting, was designed to evaluate the efficacy of GA-guided management utilizing GA tools, including the G8. This study shows that despite significant reports of satisfaction in the GA-guided management group, no significant improvements were observed in overall survival or adverse events. These findings suggest that conventional GA tools have certain limitations.

Body weight loss (BWL), a recognized frailty phenotype ^(2)^, is an important component in the definition of cancer cachexia according to the European Palliative Care Research Collaborative ^(10)^. A previous study ^(11)^ shows an association between BWL and reduced quality of life in addition to shorter survival among patients with advanced non-small cell lung cancer. A previous study ^(12)^ revealed that immune checkpoint inhibitors (ICIs) alone may be preferable to ICI/chemotherapy combinations for patients with advanced lung cancer and cancer cachexia, and that the negative prognostic effect of cancer cachexia in patients treated with ICI-containing regimens requires novel interventions.

Sarcopenia, the progressive loss of muscle mass and function, is associated with adverse outcomes such as falls, frailty, and mortality ^(13)^. The Asian Working Group for Sarcopenia (AWGS) 2019 guidelines define it using physical function, handgrip strength, and muscle mass ^(14)^ using either dual-energy X-ray absorptiometry or bioelectrical impedance analysis (BIA). Patients with lung cancer with sarcopenia often have reduced responsiveness to pharmacotherapy ^(15), (16), (17)^.

BIA is widely used across various fields, including nutritional assessment, as a practical method for estimating body composition by measuring the body’s impedance to a low-intensity alternating current. Among BIA-derived parameters, phase angle (PhA) is directly measured without using estimation equations and is considered an indicator of cell membrane functional integrity ^(18), (19)^. PhA predicts overall survival in patients with cancer ^(20)^. However, PhA is significantly higher in young individuals than in older adults, and in men than in women ^(21)^, with an unclear cut-off value (CV) ^(22)^. Conversely, the extracellular water-to-total body water ratio (ECW/TBW), another BIA-derived parameter that is reportedly negatively correlated with PhA ^(23)^, may be more useful, given its CV of 0.4 is constant ^(24)^ across age and sex groups. ECW/TBW value ≥0.4 has been reported to be significantly associated with immune-related adverse events and with the duration of successful treatment using ICIs in non-small cell lung cancer suggesting its potential utility as a novel biomarker ^(25), (26)^.

The Global Leadership Initiative on Malnutrition (GLIM) criteria ^(27)^ are a new framework for diagnosing malnutrition. They incorporate metrics such as body mass index (BMI) and BWL, in addition to skeletal muscle mass index (SMI), which is a BIA parameter.

This study evaluates the associations of G8 score (G8s), BWL, and BIA parameters (including ECW/TBW and SMI) with patient characteristics, physical function, and overall survival in patients aged ≥65 years with lung cancer. Secondarily, we determine whether combining G8, BWL, and BIA parameters achieves a comprehensive GA, similarly to the approach of the GLIM criteria ^(27)^.

## Materials and Methods

### Study design

This single-center retrospective study was approved by the institutional review board of Ishikiseiki Hospital on May 17, 2025 (approval number: # 23-17) and was conducted in accordance with the principles of the 1964 Declaration of Helsinki and its subsequent amendments. Informed consent was waived owing to the retrospective design and data anonymization. The study adhered to the opt-out policy of the hospital (https://www.ishikiriseiki.or.jp/patient/optout/).

### Patient selection

Participants were selected if they met the following criteria: aged ≥65 years with a diagnosis of lung cancer who were hospitalized between March 1, 2023 and December 31, 2024; underwent G8 screening at Ishikiriseiki Hospital during outpatient treatment planning or after hospitalization; underwent a physical function test and physical therapy on admission; underwent BIA before treatment; and experienced patient-reported or objectively verifiable weight change within 6 months.

### Clinical parameters

Baseline data were obtained from the clinical records of patients, including age, sex, Eastern Cooperative Oncology Group performance status (ECOG PS), histology subtype (adenocarcinoma/squamous cell carcinoma/small cell lung cancer/others), disease stage (II-III/IV/recurrence), BWL >5%, and smoking status (never/former/current). BMI (kg/m^2^) was calculated as weight (kg) divided by height squared (m^2^).

### G8 screening

The G8 screening form comprises a questionnaire (translated from the original ^(6)^ into Japanese ^(28)^) and basic patient data: screening date, age, sex, smoking status (pack-years), ECOG PS [0-4], maximum handgrip strength (MHGS, kg), BMI (kg/m^2^), and treatment plan.

### Physical function tests

Physical therapists conducted the following tests during hospitalization: maximum lower leg calf circumference (MLLCC, cm); MHGS (kg) measured using a GT-1201-D dynamometer (OG Wellness Co., Ltd., Okayama, Japan ); 4-meter gait speed (4MGS, m/s) measured twice over a 4-m distance using a TD-392 stopwatch (Tanita Co., Ltd., Tokyo, Japan); and five times sit-to-stand test (5TSTS, s) measured using a TD-392 stopwatch (Tanita Co., Ltd., Tokyo, Japan), requiring five repetitions from a 40-cm chair. The Short Physical Performance Battery (SPPB) was performed per the National Institute on Aging protocol ^(29)^ using three components: (i) standing balance tests (participants were made to hold three different stances for 10 sec each: feet side by side, semi-tandem, and tandem), (ii) 4MGS test, and (iii) 5STS test. The final SPPB score (range 0-12) was the sum of these three components, serving as the measure of overall physical function.

### BIA and parameters

Multi-frequency (MF)-BIA was measured using the InBody970 device (InBody Japan, Tokyo, Japan) during G8 screening. Segmental resistance (the left and right upper limbs, lower limbs, and trunk) was measured using eight surface electrodes placed on the thumbs, fingers, balls of the feet, and heels, with arms abducted from the torso. Sensors connected to microprocessor-controlled switches and an impedance analyzer were used to measure segmental resistance along the z-axis at six frequencies―1 kHz, 5 kHz, 50 kHz, 250 kHz, 500 kHz, and 1,000 kHz―and reactance along the x-axis at frequencies of 5 kHz, 50 kHz, and 250 kHz.

MF-BIA was used to estimate TBW on the basis of impedance values generated as electric current flowed through the water in the body. Ph-A was calculated using resistance (R) and reactance (Xc; measured at 50 kHz) according to the equation: Ph-A (˚) = arctangent (Xc/R) × (180/π) ^(30)^.

ECW and intracellular water values were estimated on the basis of the balance between high- and low-frequency currents traveling through TBW and ECW, respectively. ECW/TBW was subsequently calculated on the basis of previously established principles ^(31)^. The SMI measured by BIA was determined as previously described ^(32)^, by dividing total skeletal muscle mass of the limbs by the square of height.

### CVs

The following CVs were applied: G8s >14.0 (better outcome) ^(33)^; reduced muscle strength defined as an MHGS <28.0 kg for men and <18.0 kg for women ^(14)^; reduced muscle mass defined by an MLLCC <34 cm for men and <33 cm for women, or an SMI <7.0 kg/m^2^ for men and <5.7 kg/m^2^ for women ^(14)^ ; low physical function defined by an SPPB score ≤9 ^(14)^, an MGS <1 m/s ^(14)^; or a 5TSTS ≥12.0 s ^(34)^; fluid imbalance indicated by ECW/TBW ≥0.4, ^(35)^ and median CVs for other factors.

### Definition of sarcopenia and cancer cachexia

On the basis of the definition of AWGS 2019 ^(14)^, sarcopenia was specified in this study as reduced muscle mass plus low muscle strength or low physical function.

Cancer cachexia was defined per the European Palliative Care Research Collaborative ^(10)^ criteria as follows: BWL >5% over the past 6 months, or BMI <20 with BWL > 2%, or sarcopenia with BWL >2%.

Definition of the severity in Frailty Using GA Combining G8 Score, weight loss, and BIA parameters

Similarly to the GLIM criteria ^(27)^, we developed a tentative frailty assessment method combining G8s, BWL, SMI, and ECW/TBW, and BIA parameter. In the first stage, patients were classified as none frailty if their G8s >14.0. In the second stage, patients with a G8s ≤14.0 were classified as mild frailty if they had neither SMI < CV nor BWL >5%, and as moderate frailty if they had either or both. In the third stage, using ECW/TBW previously reported as a frailty biomarker, ^(26)^, the frailty level was reduced by one rank (e.g., none became mild) if their ECW/TBW ≥0.4.

### Statistical analyses

Correlations were conducted using Spearman’s rank correlation coefficient, with scatterplots and regression lines. Group comparisons were evaluated using Fisher’s exact test or the Mann-Whitney U test. Logistic regression analysis was performed as a multivariate analysis to confirm the significance of factors identified in the univariate analysis.

Overall survival (OS) was defined as the duration from cancer treatment initiation or G8 screening to death or last follow-up. OS data were updated on April 30, 2025. OS curves were compared using the Kaplan-Meier method. Survival was analyzed using the Cox proportional hazards model and presented as hazard ratios and 95% confidence intervals (CI). Statistical significance was set at p < 0.01. All statistical analyses were conducted using R software (version 4.1.1; R Foundation for Statistical Computing, Vienna, Austria).

## Results

### Patients

In this study, 120 patients were included with the following characteristics: sex (male/female): 82/38; age (<76/≥76 years): 57/63; smoking status (smoking/non-smoking): 94/26; comorbidity count (<2/≥2): 54/66; performance status (0-1/2-3): 107/13; lung cancer histology (small cell/non-small cell/other): 19/97/4; BMI (<22.2/≥22.2 kg/m^2^): 59/61; and treatment options (surgery/anti-cancer drug/radiation alone/chemoradiation/best supportive care): 43/62/4/5/6.

### Number of patients per G8 item

The patient distribution for G8 items was as follows: A (food decrease) 4/20/96 (severe/moderate/no); B (weight loss) 27/1/20/72 (>3%/does not know/between 1 and 3 kg/no weight loss); C (mobility) 3/6/111 (bed/out-but-in/out); E (neuropsychological) 0/6/114 (severe/mild/none); F (BMI) 17/26/28/49 (<19/19 to <21/21 to <23/≥23); H (>3 drugs) 75/45 (yes/no); P (self-rated health) 32/10/36/42 (not as good/unknown/as good/better); and age 11/30/79 (age >85/80-85/<80).

### Association of PhA with SMI and ECW/TBW

**Figure 1.**
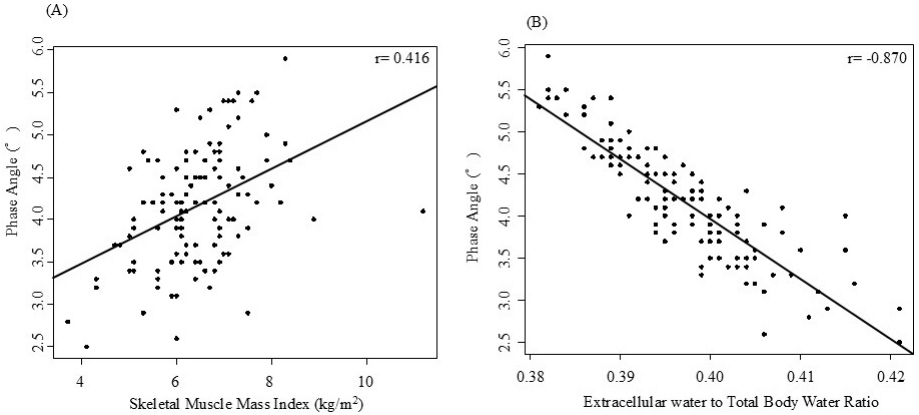
Association of PhA with SMI and ECW/TBW. (A) Scatter plot showing the relationship between PhA and SMI with R = 0.4341. (B) Scatter plot showing the inverse relationship between PhA and ECW/TBW with R = −0.8797. Abbreviations: ECW/TBW: extracellular water-to-total body water ratio; PhA: phase angle; SMI: skeletal muscle mass index.

PhA showed a significant correlation with SMI (Figure 1A) and inversely correlated with ECW/TBW (Figure 1B).

### Association of patient characteristics with G8 score, BWL, SMI, or ECW/TBW

A G8s ≤14.0 showed a weak but significant correlation with an advanced disease stage (Table 1), whereas a G8s >14.0 had a weak but significant correlation with higher BMI and surgical treatment. BWL >5% showed a weak but significant correlation with advanced stage and chemotherapy combined with ICIs, whereas BWL ≤ 5% showed a significant correlation with surgical treatment. SMI ≥CV was significantly correlated with higher BMI. ECW/TBW ≥0.4 had a weak but significant correlation with older age, and ECW/TBW <0.4 had a significant correlation with a better PS.

**Table 1.** Associations between Patient Characteristics and G8, BWL, SMI, or ECW/TBW.

	Count (%) or median (IQR)								
	Total	Geriatric-8		Body weight loss		Skeletal muscle mass index		Extracellular water-to-total body weight ratio	
		≤14	>14	≤5%	>5%	Below cut-off value	Cut-off value or higher	<0.4	≥0.4
No. of patients	120	86	34	94	26	74	46	75	45
Sex: Female	38 (31.7)	24 (27.9)	14 (41.2)	32 (34.0)	6 (23.1)	20 (27.0)	18 (39.1)	24 (32.0)	14 (31.1)
Median age (years)	76.0 (72.0−81.0)	76.0 (73.0-81.8)	75.0 (71.3-79.0)	76.0 (72.0−89.0)	75.5 (73.0−80.0)	77.0 (73.0-82.0)	75.0 (71.3-79.0)	75.0 (71.0−79.0)	80.0* (74.0−83.0)
≥76.0 age	63 (52.5)	47 (54.7)	16 (47.1)	50 (53.2)	13 (50.0)	43 (58.1)	20 (43.5)	34 (45.3)	29 (64.4)
Smoking status: ever	94 (78.3)	71 (82.6)	23 (67.6)	71 (75.5)	23 (88.5)	60 (81.1)	34 (73.9)	62 (82.7)	32 (71.1)
No. of Comorbidities	2.0 (1.0-3.0)	2.0 (1.0-3.0)	1.0 (1.0-3.0)	2 (1-3)	2 (0-3)	2 (0-3)	2 (1-3)	2 (1.0-3.0)	2.0 (0.0-3.0)
Performance status: 0-1	107 (89.2)	74 (86.0)	34 (100.0)	85 (90.4)	22 (84.6)	65 (87.8)	42 (91.3)	74 (98.7)**	33 (73.3)
Histology: other than small cell lung cancer	101 (84.2)	73 (84.9)	28 (82.4)	83 (88.3)	18 (69.2)	62 (83.8)	39 (84.8)	61 (81.3)	40 (88.9)
Stage: IV or relapse	57 (47.5)	48 (55.8)*	9 (26.5)	38 (40.4)	19 (73.1)*	38 (51.4)	19 (41.3)	35 (46.7)	22 (48.9)
Body mass index	22.3 (20.5-24.7)	21.5 (20.0-24.2)	23.4 (21.8-25.3)*	22.5 (20.5-24.9)	21.2 (20.0-23.7)	20.9 (19.8-22.5)	24.8 (22.8-26.8)**	22.3 (20.7-24.8)	21.6 (20.0-23.9)
Body mass index: ≥22.3	61 (50.8)	37 (43.0)	24 (70.6)*	52 (55.3)	9 (34.6)	24 (32.4)	37 (80.4)**	41 (54.7)	20 (44.4)
Treatment									
Surgery	45 (37.5)	25 (29.1)	20 (58.8)*	43 (45.7) **	2 (7.7)	26 (35.1)	19 (41.3)	30 (40.0)	15 (33.3)
Radiation alone	2 (1.7)	2 (2.3)	0 (0.0)	1 (1.1)	1 (3.8)	2 (2.7)	0 (0.0)	0 (0.0)	2 (4.4)
Chemoradiation therapy	5 (4.2)	4 (4.7)	1 (2.9)	3 (3.2)	2 (7.7)	3 (4.1)	2 (4.3)	5 (60.7)	0 (0.0)
Chemotherapy alone	21 (17.5)	17 (19.8)	4 (11.8)	17 (18.1)	4 (15.4)	13 (17.6)	8 (17.4)	10 (13.3)	11 (24.4)
Targeted therapy	10 (8.3)	8 (9.3)	2 (5.9)	8 (8.5)	2 (7.7)	5 (6.8)	5 (10.9)	5 (6.7)	5 (11.1)
ICI alone	3 (2.5)	1 (1.2)	2 (5.9)	2 (2.1)	1 (3.8)	2 (2.7)	1 (2.2)	1 (1.3)	2 (4.4)
Chemotherapy combined with ICI	17 (14.2)	14 (16.3)	3 (8.8)	9 (9.6)	8 (30.8)**	12 (16.2)	5 (10.9)	13 (17.3)	4 (8.9)
Chemotherapy combined with Bev, Neci, or Ram	11 (9.2)	10 (11.6)	1 (2.9)	7 (7.4)	4 (15.4)	6 (8.1)	5 (10.9)	9 (12.0)	2 (4.4)
BSC	6 (5.0)	5 (5.8)	1 (2.9)	4 (4.3)	2 (7.7)	5 (6.8)	1 (2.2)	2 (2.7)	4 (8.9)

Abbreviations: IQR, interquartile range; ICI, immune checkpoint inhibitors; Bev, bevacizumab; Neci, necitumumab; Ram, ramcirumab; BSC, best supportive care *p<0.01, **p<0.001

### Associations of physical function with G8 Score, BWL, SMI, or ECW/TBW

The G8s had no significant correlation with any measure of physical function (Table 2). However, BWL ≤5% showed a weak but significant correlation with higher MLLCC. SMI ≥CV was significantly associated with higher MLLCC and MHGS. Moreover, ECW/TBW <0.4 was significantly correlated with higher MHGS, 4MGS ≥1 m/s, and 5TSTS <12.0 s.

**Table 2.** Associations between Physical Functions and G8, BWL, SMI, or ECW/TBW.

	Count (%) or median (IQR)								
	Total	Geriatric-8		BWL		SMI		ECW/TBW	
		≤14	>14	≤5%	>5%	< CV	≥CV	<0.4	≥0.4
Number of patients	120	86	34	94	26	74	46	75	45
Maximum grip strength: cut-off value or higher	56 (46.7)	36 (41.9)	20 (58.8)	42 (44.7)	14 (53.8)	25 (33.8)	31 (67.4)**	45 (60.0) **	11 (24.4)
Maximum lower leg calf circumference: cut-off value or higher	39 (32.5)	22 (25.6)	17 (50.0)	36 (38.3)*	3 (11.5)	10 (13.5)	29 (63.0)**	26 (34.7)	13 (28.9)
Gait speed: ≥1.0m/s	67 (55.8)	44 (51.2)	23 (67.6)	55 (58.5)	12 (46.2)	39 (52.7)	28 (60.9)	55 (73.3)**	12 (26.7)
Five times sit to stand test: <12s	77 (64.2)	53 (61.6)	24 (70.6)	60 (63.8)	17 (65.4)	48 (64.9)	29 (63.0)	57 (76.0)**	20 (44.4)

Abbreviations: G8, Geriatric-8; BWL, body weight loss; SMI, skeletal muscle mass index; ECW/TBW, extracellular-to-total body water ratio; *p<0.01, **p<0.001

### Associations of neuropsychological function with G8 Score, BWL, SMI, or ECW/TBW

In item E of the G8, which represents neuropsychological function, six patients had mild dementia. Of these, six had G8 ≤14.0 and SMI < CV; five had ECW/TBW ≥0.4, and none had BWL >5%.

### Correlations among G8 Score, BWL, SMI, or ECW/TBW

A G8s ≤14.0 was significantly correlated with BWL >5% (*p* < 0.0001) and ECW/TBW ≥0.4 (*p* = 0.0061), and tended to correlate with SMI < CV (*p* = 0.0211). BWL >5% tended to correlate with SMI < CV (*p* = 0.0247). No significant correlations were observed for other factors.

### Association of cancer cachexia, sarcopenia, or physical function decrease with G8 Score, BWL, SMI, or ECW/TBW

Table 3 presents the associations of cancer cachexia, sarcopenia, or physical dysfunction with G8s, BWL >5%, SMI, or ECW/TBW in the univariate analysis. A G8s ≤14.0 had a weak but significant correlation with sarcopenia and a significant correlation with cancer cachexia. BWL >5% was significantly associated with cancer cachexia. SMI < CV showed a weak but significant correlation with sarcopenia and cancer cachexia. ECW/TBW ≥0.4 was significantly associated with sarcopenia and physical function decrease. Furthermore, although not shown in Table 3, five of the six patients with mild dementia on the E item of the G8 had SPPB ≤9.

**Table 3.** Correlations between Cancer Cachexia, Sarcopenia or Physical Function Decrease and Geriatric-8, Body Weight Loss, Skeletal Muscle Mass Index, or ECW/TBW Based on Univariate Analysis.

			Count (%)		
		N	Cancer Cachexia	Sarcopenia	Physical Function Decrease^§^
Geriatric-8	≤14.0	86	39 (45.3)**	66 (76.7)*	28 (32.6)
	>14.0	34	1 (2.9)	17 (50.0)	4 (11.8)
Body weight loss	≤5%	94	14 (14.9)	64 (68.1)	23 (24.5)
	>5%	26	26 (100)**	19 (73.1)	9 (34.6)
Skeletal muscle mass index	< CV	74	33 (44.6)*	58 (78.4)*	21 (28.4)
	≥CV	46	7 (15.2)	25 (54.3)	11 (23.9)
ECW/TBW	<0.4	75	22 (29.3)	40 (53.3)	9 (12.0)
	≥0.4	45	18 (40.0)	43 (95.6)**	23 (51.1) **

^§^ Physical function decrease was represented by the short physical performance battery ≤9. Abbreviations: ECW/TBW: extracellular-to-total body water ratio *p<0.01, **p<0.001

Table 4 confirmed the associations of cancer cachexia, sarcopenia, and physical function decrease with G8, BWL >5%, SMI, and ECW/TBW in the logistic regression analysis.

**Table 4.** Multivariate Analysis of Risk Relating to Cancer Cachexia, Sarcopenia, and Physical Function Decrease among G8 >14.0, BWL >5%, SMI ≥CV, and ECW/TBW ≥0.4.

	Cancer Cachexia		Sarcopenia		Physical Function Decrease^§^	
	Odds ratio (95% CI)	*p* value	Odds ratio (95% CI)	*p* Value	Odds ratio (95% CI)	*p* Value
G8 >14.0	0.04 (0, 0.28)	0.028	0.47 (0.17, 1.33)	0.154	0.47 (0.13, 1.71)	0.252
BWL >5%	-	-	0.54 (0.16, 1.89)	0.337	1.29 (0.44, 3.83)	0.643
SMI ≥CV	0.22 (0.09, 0.56)	0.032	0.27 (0.1, 0.72)	0.007	1.00 (0.378, 2.66)	0.995
ECW/TBW ≥ 0.4	1.61 (0.74, 3.49)	0.746	19.64 (4.18, 92.23)	< 0.001	6.72 (2.65, 17.05)	< 0.001

^§^ Physical function decrease was represented by the short physical performance battery ≤9. Abbreviations: G8, Geriatric-8; BWL, body weight loss; SMI, skeletal muscle mass index; ECW/TBW, extracellular-to-total body water ratio; CI, confidence interval; CV, cut-off value.

Because BWL >5%, which is a criterion for cancer cachexia, was too strongly correlated with cancer cachexia, it was excluded from multivariate analysis of cancer cachexia. Cancer cachexia was weakly associated with G8, and SMI. sarcopenia was strongly associated with ECW/TBW ≥0.4 and SMI < CV. Physical function decrease was strongly associated with ECW/TBW ≥0.4 only.

### OS according to the G8 Score, BWL, SMI, and ECW/TBW

**Figure 2.**
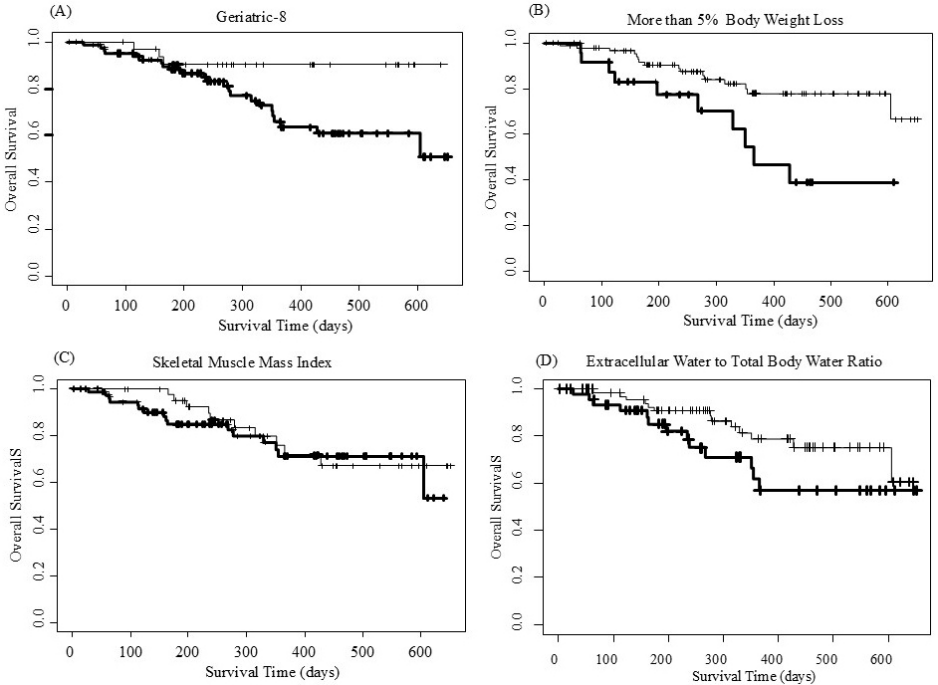
Overall survival according to G8 score, BWL, SMI, and ECW/TBW. The thin lines and solid lines show (A) patients with G8 >14.0 and those with G8 ≤14.0, (B) patients with BWL ≤5% and those with BWL >5%, (C) patients with SMI ≥CV and those with SMI < CV, and (D) patients with ECW/TBW <0.4 and those with ECW/TBW ≥0.4, respectively. BWL: body weight loss; CV: cut-off value; ECW/TBW: extracellular water-to-total body water ratio; G8: Geriatric-8; SMI: skeletal muscle mass index.

Figure 2 and Table 5 present a comparison of OS in G8s ≤14.0, BWL >5%, SMI < CV, or ECW/TBW ≥0.4, and counterparts. Figure 2A reveals that patients with a G8s >14.0 had a significantly longer survival than those with lower scores. Figure 2B shows that patients with BWL >5% had a significantly shorter survival duration than those with BWL ≤5%. No significant difference in survival duration was observed between patients with SMI ≥CV and those below the cutoff (Figure 2C). Figure 2D shows that patients with ECW/TBW ≥0.4 tended to have a shorter survival duration than those with lower ratios; however, the difference was not statistically significant.

**Table 5.** Comparison of Overall Survival in Patients Aged ≥65 Years with Lung Cancer in Geriatric-8 ≤14.0, Body Weight Loss >5%, Skeletal Muscle Mass Index < CV, or ECW/TBW ≥0.4, and Counterparts.

		N	Events	Median (days) (95% CI)	Hazard Ratio (95% CI)	*p* Value
Geriatric-8	≤14.0	86	23	NA (428, NA)	0.2938 (0.0882, 0.9790)	0.0461
	>14.0	34	3	NA (NA, NA)		
Body weight loss	≤5%	94	16	NA (605, NA)	2.8805 (1.3040, 6.3600)	0.0089
	>5%	26	10	366 (329, NA)		
Skeletal muscle mass index	≤CV	74	16	NA (605, NA)	0.8352 (0.3786, 1.8420)	0.6550
	>CV	46	10	NA (NA, NA)		
ECW/TBW	<0.4	75	13	NA (605, NA)	1.9360 (0.8962, 4.1820)	0.0927
	≥0.4	45	13	NA (355, NA)		

Abbreviations: CV, cut-off value; ECW/TBW, extracellular-to-total body water ratio; CI, confidence interval; NA, not available.

### Algorithm of GA according to the G8 Score, BWL, SMI, or ECW/TBW

**Figure 3.**
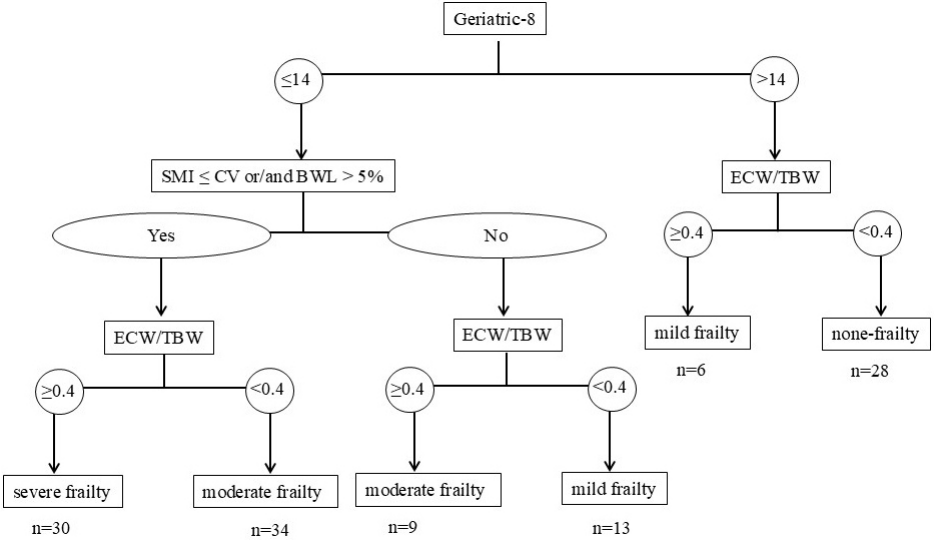
Algorithm of geriatric assessment according to G8 score, BWL, SMI, and ECW/TBW. Of the 120 patients analyzed, the distribution across the frailty categories was as follows: 28 patients were classified as none, 19 as mild, 43 as moderate, and 30 as severe. BWL: body weight loss; CV: cut-off value; ECW/TBW: extracellular water-to-total body water ratio; G8: Geriatric-8; GA: geriatric assessment; SMI: skeletal muscle mass index.

A G8s >14.0 was negatively associated with cancer cachexia. BWL >5% indicates the presence of cancer cachexia. ECW/TBW ≥0.4 shows sarcopenia and physical function decrease. SMI below the CV implies a tendency to cancer cachexia and sarcopenia. As shown in Figure 3, based on the characteristics of these four factors, an algorithm was developed. Of the 120 patients analyzed, the distribution across the frailty categories was as follows: 28 patients were classified as none, 19 as mild, 43 as moderate, and 30 as severe.

### OS according to frailty categories

**Figure 4.**
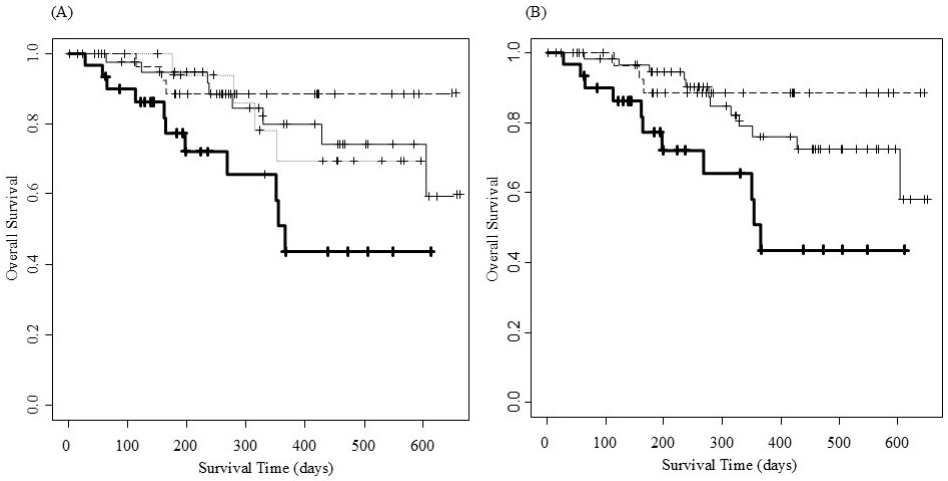
Overall survival curves according to frailty categories. (A) shows overall survival curves for frailty: none (dashed line), mild (dotted line), moderate (thin line), and severe (solid line), respectively. (B) shows overall survival curves for frailty: none (dashed line), mild combined with moderate (thin line), and severe (solid line), respectively.

Figure 4A presents the OS curves and data (n/events/median/[days]/95% CI) using four categories of frailty (none, mild, moderate, and severe) as 28/3/not available (NA)/NA, 19/4/NA/352-NA, 43/8/NA/605-NA, and 30/11/366/268-NA, respectively. Figure 4B shows the OS curves and data (n/events/median/[days]/95% CI) using three frailty groups (none, mild combined with moderate, severe) as 28/3/NA/NA, 62/12/NA/605-NA, and 30/11/366/268-NA, respectively.

## Discussion

To our knowledge, this study is the first to show concurrent associations between the G8s, BWL, SMI, and ECW/TBW with patient characteristics and physical function. Each of these four factors exhibited distinct correlations with patient characteristics and physical function. Specifically, the G8s was significantly associated with the other three indicators. SMI < CV was associated with BWL >5%; however, ECW/TBW ≥0.4 showed no correlation with BWL >5% or SMI < CV. A G8s >14.0 may help identify patients in relatively good condition. Nevertheless, because a large proportion of patients had a G8s ≤14.0, it may be challenging to assess GA using the G8s alone.

Consistent with previous studies ^(11), (36)^, BWL >5% was significantly associated with malnutrition and poor survival, highlighting its clinical relevance for GA.

SMI, as measured using BIA, remains a diagnostic criterion for sarcopenia ^(14)^. In the present study, SMI showed a positive linear relationship with PhA, consistent with previous findings ^(23)^. A meta-analysis ^(37)^ suggests that the psoas muscle index, measured through computed tomography, may be a more accurate method for diagnosing sarcopenia than BIA, which is affected by hydration. Our study reveals that although SMI is significantly associated with muscle strength and volume, it is not correlated with physical function. Further research is required to clarify the effectiveness of BIA in sarcopenia diagnosis.

Furthermore, ECW/TBW showed a negative linear relationship with PhA, consistent with previous research ^(23)^. Among the four factors evaluated, ECW/TBW was most strongly associated with physical function. Zheng et al. ^(38)^ reported that ECW/TBW ≥0.4 is associated with malnutrition and impaired physical performance. Tanaka et al. ^(39)^ also revealed that higher ECW/TBW is strongly associated with an increased risk of locomotive syndrome. In Japan, locomotive syndrome is considered a form of frailty and is characterized by an age-related physical function decrease ^(40)^. Three studies ^(38), (39)^, including the present one, show that ECW/TBW correlates with physical function decrease, supporting its use as a biomarker for detecting physical function decrease.

Older patients with cancer and cognitive impairment may face difficulty understanding their diagnosis, prognosis, and treatment options, including the associated benefits and risks ^(41)^. A systematic review ^(42)^ summarized studies on the impact of pre-treatment cognitive impairment on toxic side effects, treatment completion, and survival in older patients with cancer. The predictive role of cognitive impairment remains unclear, necessitating further study. A study reported that cognitive impairment is often overlooked during routine assessments ^(43)^. This study indicated that physicians are likely unaware of patients with cognitive impairment. In the present study, only six patients (5%) had mild dementia; as shown previously ^(43)^, the G8 assessment alone may underrecognize dementia. However, the observation that all six cases in our study had a G8s ≤14.0 and SMI < CV, and that five of these cases also had an ECW/TBW ≥ 0.4 and an SPPB score ≤9, indicates that the G8 and BIA parameters may be useful for screening individuals with cognitive impairment. Previous studies linked cognitive function to SPPB ^(44)^, and higher ECW/TBW to reduced cognitive function ^(45)^; more research is needed to determine the association between SPPB or BIA parameters and cognitive impairment.

In a study ^(46)^, Japanese citizens aged 75 years or older were found to have three or more co-occurring diseases. However, the median number of comorbidities in the present study was two, which is relatively low. This discrepancy may be partly due to the underreporting or omission of comorbidities in medical records.

Previous studies ^(47), (48), (49)^ showed GA-guided management for older patients with cancer reduced adverse events but did not significantly improve OS.

A recent randomized study ^(9)^ showed no significant improvement in OS or adverse events, despite satisfaction with GA-guided management. These studies highlight GA limitations, necessitating a new GA model such as that developed here.

In this study, using the G8 to screen for general frailty, BWL >5% to detect cancer cachexia, SMI for sarcopenia, and ECW/TBW for sarcopenia and physical function decrease, patients can be stratified into three frailty levels (none, mild combined with moderate, and severe). These may correspond to the frailty levels―fit, vulnerable, unfit―recommended by the JCOG Committee on Older Patients’ Research Policy ^(5)^. In older patients with lung cancer, GA incorporating the G8s, BWL, and BIA-derived parameters, such as those in the GLIM criteria ^(27)^, may offer a simpler, more objective alternative to traditional GA tools.

This study has some limitations. First, it was a single-center retrospective study. Second, the sample size was relatively small. Third, variability in treatment may have influenced survival outcomes. Fourth, cognitive function was not adequately assessed. Fifth, baseline data on patients, including complications, could be overlooked in retrospective studies.

Further studies are required to investigate the effectiveness of GA in combination with G8s, BWL, and BIA among patients receiving the same treatments.

## Article Information

### Acknowledgments

The authors thank Ms. Masako Tanigawa and Ms. Hiroko Satoh (Clinical Research Support Center, Ishikiriseiki Hospital, Japan), who assisted with data collection and data organization. Finally, the authors thank Editage (www.editage.jp) for English language editing.

### Author Contributions

Tomonori Hirashima, Naoki Yoshimoto, Kenichi Minami, and Kazuto Hirata were involved in the conception and design of the study. Tomonori Hirashima, Naoki Yoshimoto, Yoshitaka Fujii, Akira Todoriki, Erika Matsui, Atsuhito Hikiishi, Kenji Nakahama, Eriko Tani, Satoru Yamamoto, Nobuhiro Izumi, and Kenichi Minami performed G8 screening and participated in the acquisition of clinical and laboratory data. Yuhki Takahashi, Masaki Ninomiya, Keita Mizukoshi, Sora Obata, and Yusuke Watari evaluated physical function tests and instructed the patients in self-exercise. Tomonori Hirashima, Naoki Yoshimoto, and Hidekazu Suzuki participated in statistical analysis. All authors read and approved the final manuscript.

### Conflicts of Interest

None

### Informed Consent

The requirement for consent was waived owing to the study’s retrospective design and anonymization of data. This study was also conducted in accordance with the hospital’s opt-out policy (https://www.ishikiriseiki.or.jp/patient/optout/).

### IRB Approval Code and Name of the Institution

This study was approved by the institutional review board of Ishikiseiki Hospital on

May 17, 2025 (approval number: # 23-17).
